# Knockdown of TFAM in Tumor Cells Retarded Autophagic Flux through Regulating p53 Acetylation and PISD Expression

**DOI:** 10.3390/cancers12020493

**Published:** 2020-02-20

**Authors:** Xu Jiang, Jun Wang

**Affiliations:** 1 Key Laboratory of High Magnetic Field and Ion Beam Physical Biology, Chinese Academy of Sciences, Hefei 230031, China; jx089254@mail.ustc.edu.cn; 2 Graduate School of the University of Science and Technology of China, Hefei 230026, China

**Keywords:** TFAM, p53, p53 acetylation, PISD, LC3-II, autophagy

## Abstract

Mitochondrial transcription factor A (TFAM) is required for mitochondrial DNA replication and transcription, which are essential for mitochondrial biogenesis. Previous studies reported that depleting mitochondrial functions by genetic deletion of TFAM impaired autophagic activities. However, the underlying mechanisms remain largely unknown. In the current study, we identified that knockdown of TFAM repressed the synthesis of autophagy bio-marker LC3-II in tumor cells and decreased the expression of phosphatidyl-serine decarboxylase (PISD). Besides, downregulation of PISD with siRNA reduced the level of LC3-II, indicating that depletion of TFAM retarded autophagy via inhibiting PISD expression. Furthermore, it was found that the tumor repressor p53 could stimulate the transcription and expression of PISD by binding the *PISD* enhancer. Additionally, the protein stability and transcriptional activity of p53 in TFAM knockdown tumor cells was attenuated, and this was associated with decreased acetylation, especially the acetylation of lysine 382 of p53. Finally, we identified that TFAM knockdown increased the NAD^+^/NADH ratio in tumor cells. This led to the upregulation of Sirtuin1 (SIRT1), a NAD-dependent protein deacetylase, to deacetylate p53 and attenuated its transcriptional activation on PISD. In summary, our study discovered a new mechanism regarding disturbed autophagy in tumor cells with mitochondrial dysfunction due to the depletion of TFAM.

## 1. Introduction 

Mitochondrion is essential for cell homeostasis and is involved in the tricarboxylic cycle, lipids’ biosynthesis, inflammation, aging, and apoptosis [1,2,3]. Human mitochondrial transcription factor A (TFAM) is encoded by the genomic DNA and is required for the replication and transcription of mitochondrial DNA, which are essential for mitochondrial biogenesis [4,5]. Depletion of TFAM causes dysfunctions in the respiratory chain and inhibits the fusion of lysosomes with autophagosomes, thereby resulting in impaired autophagic flux [6]. Other reports also demonstrated the connection between mitochondria and lysosome, and the reasons how the damaged mitochondria led to the inhibition of autophagy [7,8]. However, the underlying mechanisms remain largely to be explored.

Autophagy is a highly evolutionarily conserved mechanism for the recycling and degradation of cytoplasmic constituents [9]. Autophagosomes engulf their cargo and constitute a closed structure with a double membrane, then fuse with lysosomes, where the substrates are hydrolyzed and recycled [10]. The lipidation of LC3 drives the growth and the closure of the autophagosome [11]. This process needs ATG7, ATG3, and the ATG5–ATG12-ATG16L1 conjugate, which function as E1-like, E2-like, and E3 ubiquitin ligase, respectively, to transfer the phosphatidylethanolamine (PE) to LC3 [12]. One of the major pathways for PE synthesis in mammalian cells is utilizing PISD (phosphatidyl-serine decarboxylase), an enzyme located in the inner membrane of mitochondria, to produce PE by decarboxylating phosphatidylserine (PS) [13]. PE is ultimately exported to other organelles to perform its biological functions. PE deficiency influences mitochondrial morphologies and oxidative phosphorylation. Disruption of the *Pisd* gene in mouse results in embryonic lethality [14,15]. Overexpression of the PE-generating enzyme *Psd1* or artificially increasing intracellular PE levels in yeast significantly promotes autophagic flux [16]. Therefore, the mitochondrial functions and PISD are vital for sustaining autophagy in cells.

p53, as a cellular gatekeeper, plays dual roles in autophagy regulation depending on its subcellular localization [17]. In the nucleus, p53 functions as a pro-autophagic factor in transcription dependent or independent manners. p53 induces autophagy by transactivating the β1 and β2 subunits of AMPK, TSC2, PTEN, SESN 1, and SESN 2, all functionally antagonizing the mTOR pathways [18,19]. p53 also activates autophagy through damage regulated autophagy modulator (DRAM), a p53 target gene encoding a lysosomal protein that induces macro-autophagy [20]. In contrast, when located in the cytoplasm, p53 suppresses the induction of autophagy [21]. p53 harbors several conserved sites that can be acetylated [22]. Acetylation is pivotal for the functional activations of p53. Blocking the acetylation of p53 by simultaneously substituting the lysine at the sites of 120, 164, 370, 372, 373, 381, 382, and 386 with arginine completely abolishes p53-mediated cell cycle arrest and apoptosis [23]. Additionally, after treatment with short-term starvation, the acetylation of p53 in cell nucleus is increased, and this further promotes autophagy via enhancing the transcriptional activity of p53 [24].

In our previous study, we verified that the downregulation of p53 was associated with enhanced radiation sensitivity in TFAM knockdown tumor cells [25]. We therefore hypothesized that the deregulation of p53 could also affect autophagy in TFAM knockdown cells. Our present study showed that the depletion of TFAM retarded autophagy. We also found that the attenuated expression of TFAM resulted in the downregulation of p53 acetylation, which further blocked the transcription and expression of PISD and inhibited autophagy. These results give new insight into understanding the role of TFAM in regulating autophagy in tumor cells. 

## 2. Results 

### 2.1. TFAM Knockdown Inhibits the Formation of Autophagosome

**Figure 1:**
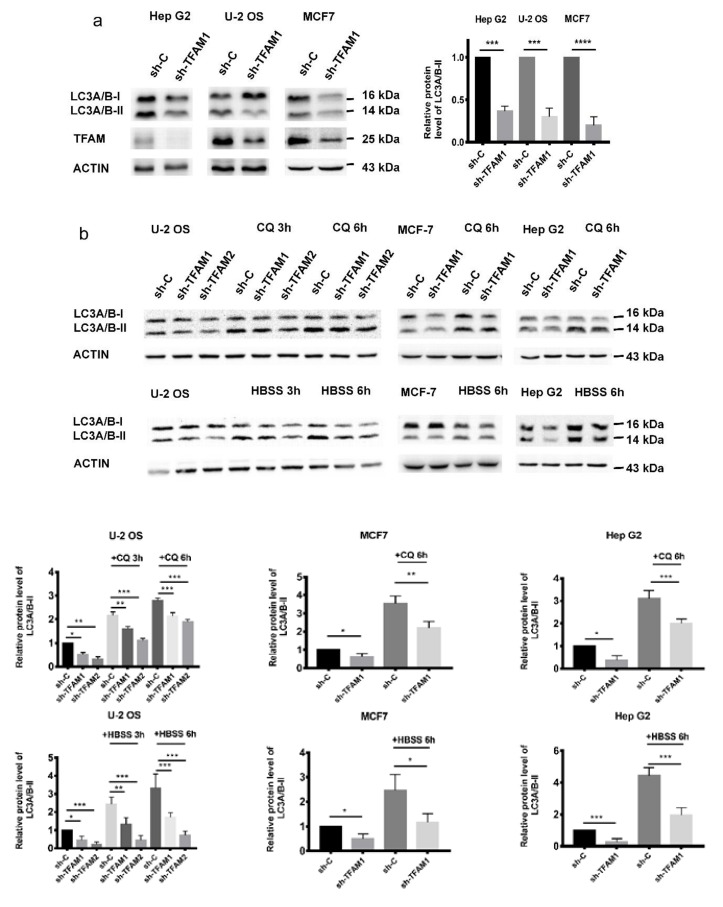

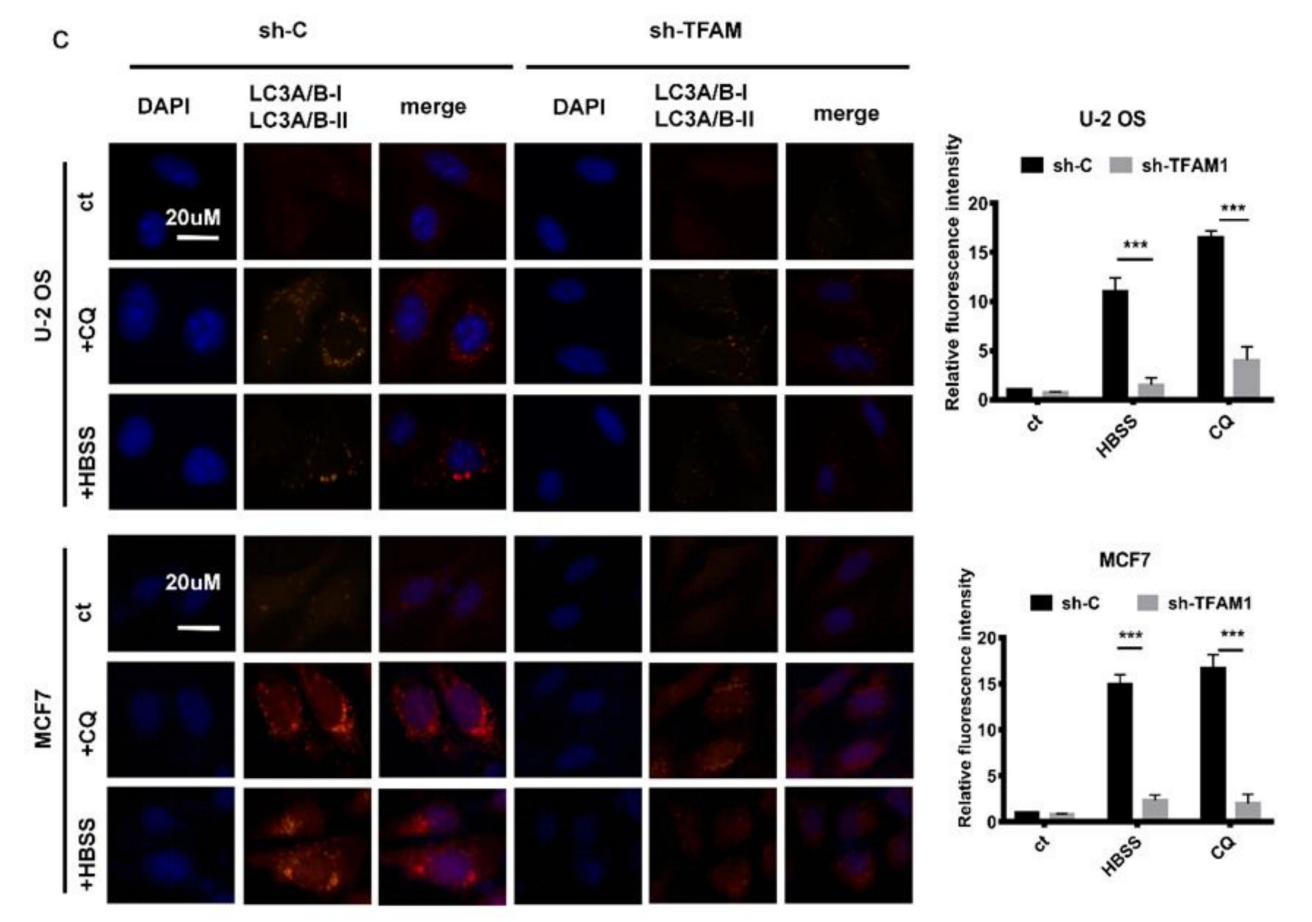
TFAM knockdown inhibits the formation of autophagosome. (**a**) Western blotting analysis of LC3-II levels in TFAM knockdown cancer cell lines. (**b**) The level of LC3-II in control shRNA (sh-C) and TFAM shRNA (sh-TFAM) transfected cells after treatment with 50 µM CQ or HBSS for three or six hours. (**c**) Immunofluorescence staining of LC3 puncta in sh-C and sh-TFAM cells after treatment with 50 µM chloroquine (CQ) or HBSS for six hours. Each independent experiment was repeated three times or more, and data are presented as the mean ± SD. *p < 0.05, **p < 0.01, ***p < 0.001.

To detect the relationship between TFAM and tumor cell autophagy, we transfected shRNA plasmids into U-2 OS, MCF7, and Hep G2 cells to generate stable cell lines with lowered expression levels of TFAM. Then, the levels of LC3-II, a marker of autophagy, were detected. It was found that LC3-II levels were decreased in TFAM knockdown cells (Figure 1a and Figure S1).

In order to determine whether the autophagy flow was inhibited, we used chloroquine (CQ), an inhibitor that blocked the fusion of lysosome with autophagosome, to treat the cells and confirmed that TFAM knockdown indeed suppressed autophagy (Figure 1b and Figure S2). We also treated the cells with Hank’s balanced salt solution (HBSS) to mimic starvation and stimulate autophagy. As expected, both immunoblotting and immunofluorescent staining results showed that autophagy was restrained when the expression of TFAM was inhibited (Figure 1c and Figure S2). Based on the above results, we inferred that the formation of autophagosome was blocked when TFAM was downregulated.

### 2.2. Decreased PISD Results in the Inhibition of Autophagy in TFAM Knockdown Cells 

**Figure 2:**
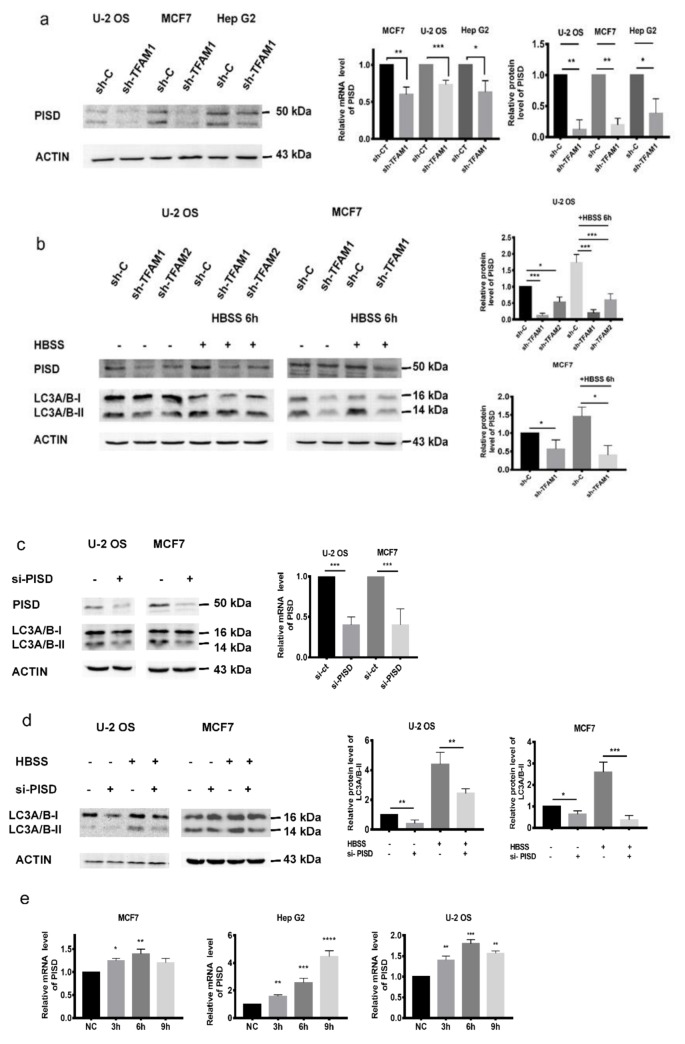

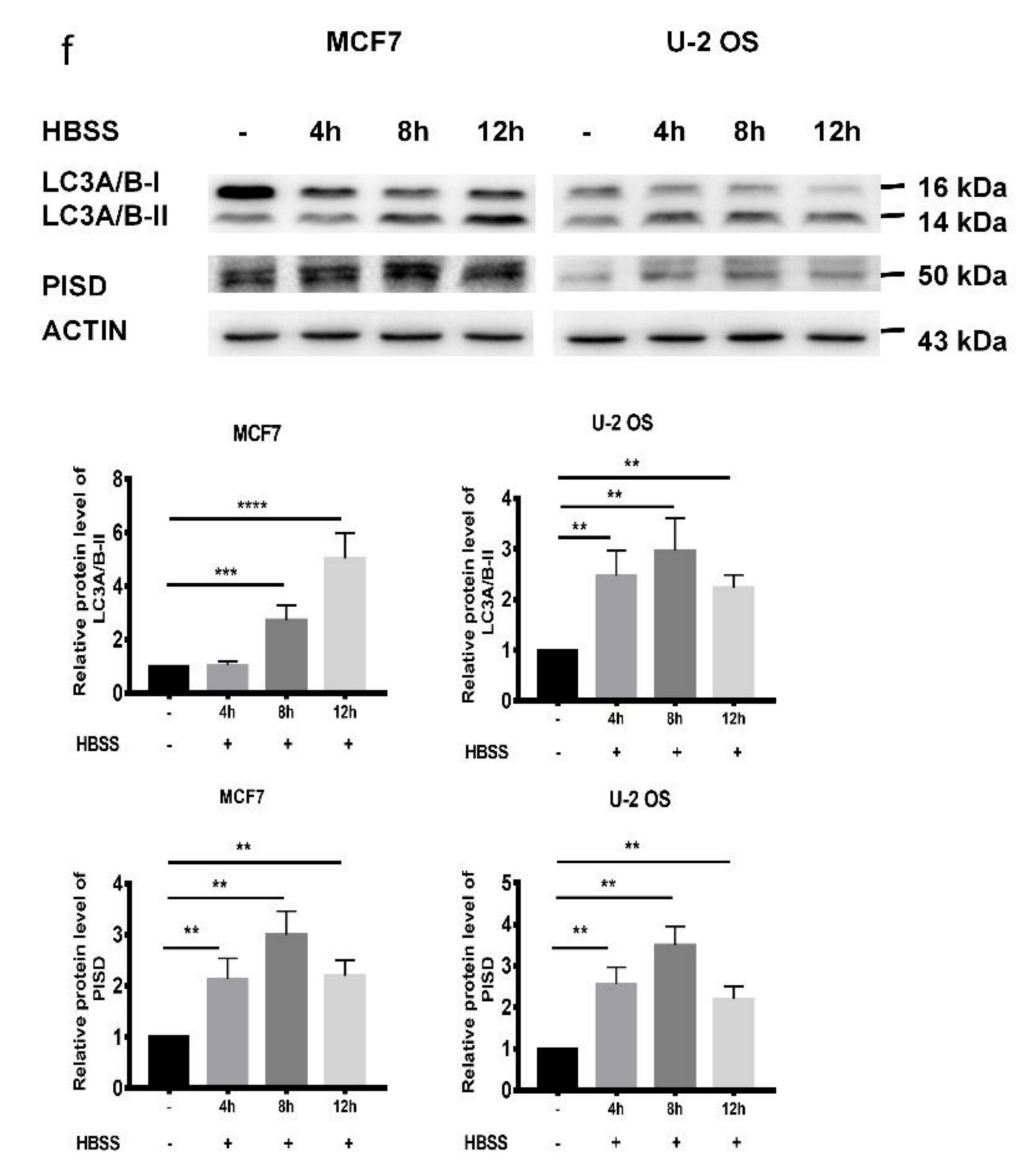
Decreased PISD results in the inhibition of autophagy in TFAM knockdown cells. (**a**) Western blotting analysis of protein levels of PISD and qRT-PCR analysis of mRNA levels of PISD in TFAM knockdown cancer cell lines. (**b**) The protein levels of LC3-II and PISD in sh-C and sh-TFAM transfected cells after treatment with HBSS for six hours. (**c**) The protein levels of LC3-II after downregulating the expression of PISD. (**d**) Under HBSS treatment, the protein levels of LC3-II in U-2 OS and MCF7 cells after downregulating the expression of PISD. (**e**) The mRNA levels of PISD in tumor cells after treatment with HBSS for 3, 6, and 9 hours. (**F**) The protein levels of PISD and LC3-II in tumor cells after treatment with HBSS for 4, 8, and 12 hours. Each independent experiment was repeated three times or more, and data are presented as the mean ± SD. *p < 0.05, **p < 0.01, ***p < 0.001.

Since the retardation of autophagy was associated with mitochondria, we guessed that there might be a mitochondria-located autophagy regulator whose function could be influenced by *TFAM*. PISD is located in the mitochondrial inner membrane and important for sustaining autophagy. We found that inhibition of TFAM attenuated the expression and mRNA levels of *PISD* (Figure 2a). Besides, protein levels of PISD remained almost unchanged when the TFAM knockdown cells were treated with HBSS. However, in the control cells, HBSS treatment induced the expression of PISD (Figure 2b). Due to its function in the biosynthesis of phosphatidylethanolamine (PE), we further tested whether PISD was associated with autophagy. It was found that the LC3-II levels were reduced after the expression of PISD was inhibited by its siRNA (Figure 2c). Under HBSS treatment, LC3-II was still reduced after interfering with PISD expression (Figure 2d). However, in U-2 OS, Hep G2, and MCF7 cells, with the elongation of HBSS treatment, the mRNA levels of *PISD* and the protein levels of LC3-II showed an apparently increasing tendency (Figure 2e,f). Based on these results, we concluded that PISD was key for autophagy flux in tumor cells, and its downregulation in TFAM knockdown tumor cells resulted in the inhibition of autophagy.

### 2.3. PISD Expression is Regulated by p53

**Figure 3:**
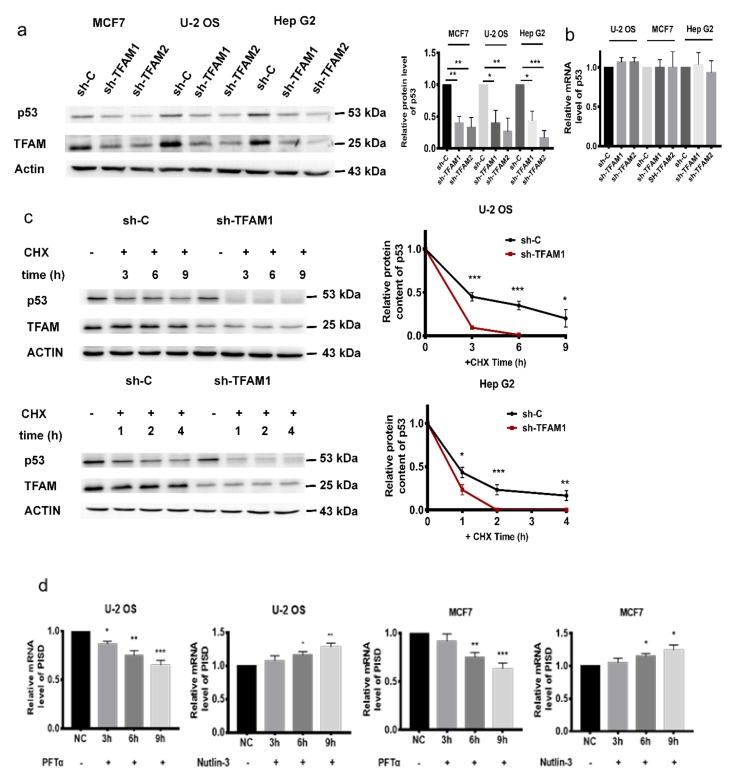

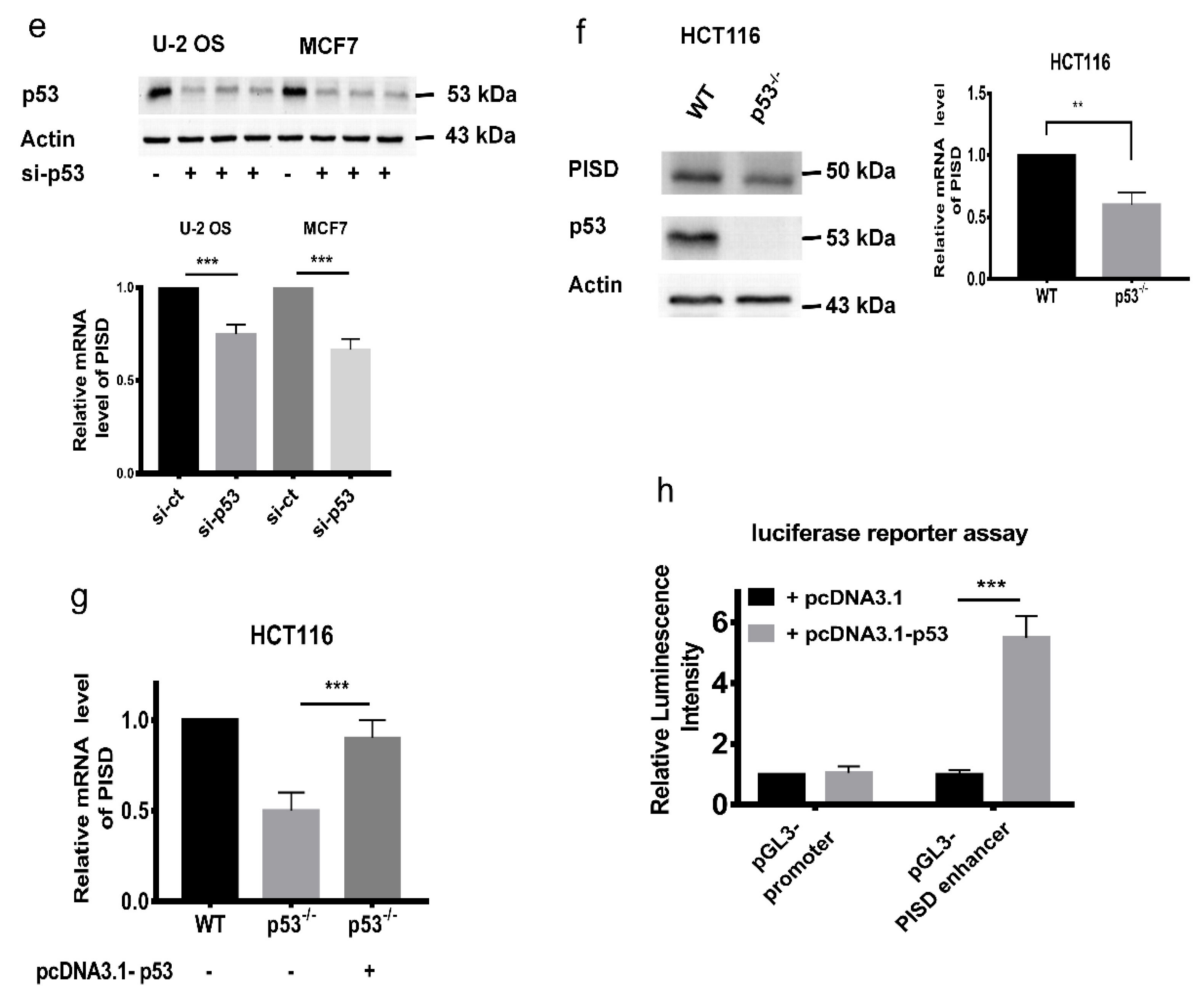
PISD expression is regulated by p53. (**a**) Western blotting analysis of protein levels of p53 in TFAM knockdown cells. (**b**) qRT-PCR analysis of p53 mRNA levels in sh-C or sh-TFAM transfected cells. (**c**) Protein stability analysis of p53 by detecting the residual levels of p53 at different time points after cycloheximide (CHX) (30 µg/mL) treatment in TFAM knockdown cells. (**d**) The mRNA levels of PISD after treatment with 10 µM pifithrin-α or 10 µM nutlin-3 respectively for 3, 6, and 9 hours in MCF7 and U-2 OS cells. (**e**) The mRNA levels of PISD in MCF7 and U-2 OS cells after the expression of p53 was inhibited by siRNA. (**f**) The protein and mRNA levels of *PISD* in HCT116 and HCT116 *p53^-/-^* cells. (**g**) The mRNA level of *PISD* in HCT116 *p53^-/-^* cells transfected with pcDNA3.1-p53. (**h**) Relative luminescence intensity in HCT116 *p53*^-/-^ cells co-transfected with the pGL3-promoter or pGL3-PISD enhancer with pcDNA3.1 and pcDNA3.1-p53, respectively. Each independent experiment was repeated three times or more, and data are presented as the mean ± SD. *p < 0.05, **p < 0.01, ***p < 0.001.

After confirming that the decreased expression of PISD in *TFAM* knockdown cells was one reason for the retarded autophagy, we then looked for the upstream regulator of *PISD*. Our previous study verified that the downregulation of p53 was associated with enhanced radiation sensitivity in TFAM knockdown tumor cells [25]. In addition to PISD, we found that in TFAM knockdown cells, the expression levels of p53 were decreased, which was consistent with our previous result. However, its mRNA levels did not show obvious changes (Figure 3a,b and Figure S1). We then tested the stability of p53 protein in TFAM knockdown cells. After treating the cells with cycloheximide (CHX) to inhibit the synthesis of nascent proteins, the residual levels of p53 in TFAM knockdown cells were significantly higher than those in the control cells (Figure 3c). Considering that p53 is a transcription regulation factor, we next investigated the effect of p53 on PISD expression. U-2 OS and MCF7 cells were treated with p53 activator nutlin-3 and inhibitor pifithrin-α, respectively. It was found that the mRNA levels of *PISD* were decreased in pifithrin-α treated cells, while increased in nutlin-3 treated cells (Figure 3d). In addition, the mRNA levels of *PISD* were attenuated in cells transfected with siRNA targeting p53 (Figure 3e). Besides, in HCT 116 *p53^-/-^* cells, both mRNA and protein levels of PISD were reduced compared to those in the parental HCT 116 cells (Figure 3f). We next transfected HCT 116 *p53^-/-^* cells with pcDNA3.1 inserted with the p53 coding sequence (pcDNA3.1-p53). It was found that the mRNA level of *PISD* was recovered to a similar level observed in HCT 116 cells (Figure 3g). JASPAR software (http://jaspar.genereg.net/) predicted that the upstream region of human *PISD* (NC_000022.11:c31648029-31646950 Homo sapiens chromosome 22, GRCh38.p12) was a putative enhancer and contained conserved p53 binding sequences. We then inserted this DNA segment into pGL3-promoter luciferase reporter vector (pGL3-PISD enhancer) and co-transfected this enhancer reporter construct with pcDNA3.1-p53 into HCT 116 *p53^-/-^* cells. As shown in Figure 3h, in the cells transfected with the pcDNA3.1-p53 and pGL3-PISD enhancer, luciferase expression was significantly stimulated, verifying that p53 could bind to the enhancer region of *PISD* to augment its transcription. Since both p53 and PISD were decreased in TFAM knockdown cells, it was speculated that the downregulation of p53 in TFAM knockdown cells impaired the expression of PISD and led to the inhibition of autophagy. 

### 2.4. The Inhibition of p53 and PISD Impairs Autophagy

**Figure 4:**
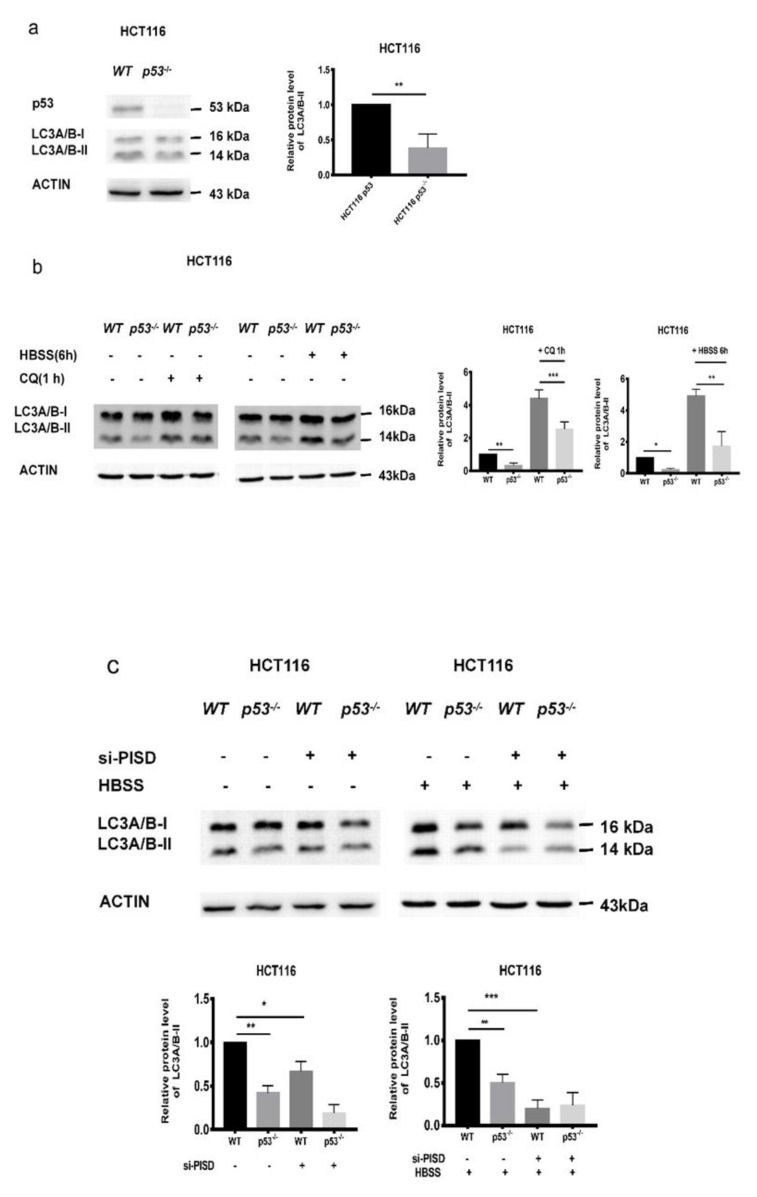

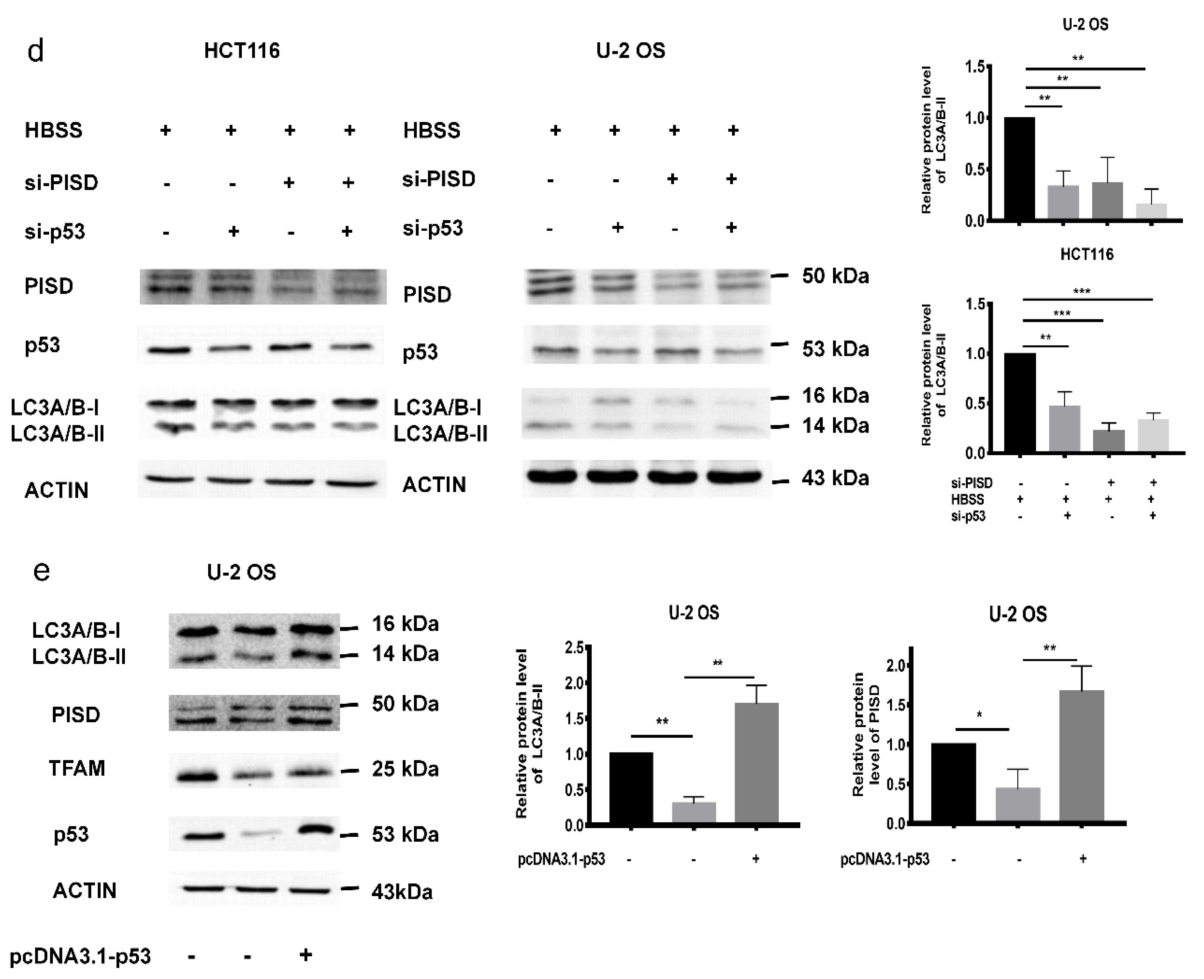
The inhibition of p53 and PISD impairs autophagy. (**a**) Protein levels of p53 and LC3-II in HCT116 or HCT116 *p53^-/-^* cells. (**b**) Protein levels of LC3-II in HCT116 *p53^-/-^* cells after treatment with CQ (50 µM) and HBSS for one and six hours, respectively. (**c**) Under HBSS treatment, the protein levels of LC3-II in HCT116 cells after downregulating PISD expression with siRNA. (**d**) Protein levels of LC3-II in HCT116 and U-2 OS cells after downregulating the expression of p53 or PISD. (**e**) The levels of LC3-II and PISD in TFAM knockdown cells after being transfected with the pcDNA3.1-p53 construct. Each independent experiment was repeated three times or more, and data are presented as the mean ± SD. *p < 0.05, **p < 0.01, ***p < 0.001.

To confirm the roles of p53 and PISD in regulating autophagy, the levels of PISD and LC3-II were detected in HCT 116 and HCT 116 *p53^-/-^* cells. Both of them were decreased in HCT 116 *p53^-/-^* cells (Figure 4a). In addition, after treatment with HBSS or CQ, LC3-II levels in HCT 116 *p53^-/-^* cells were lower compared to those in the corresponding HCT 116 cells (Figure 4b), indicating that p53 promoted the autophagy flux. On the other hand, under the treatment of HBSS or not, after knocking down the expression of PISD in HCT 116 and HCT 116 *p53^-/-^* cells, we observed a further reduction of LC3-II levels (Figure 4c). These indicated that the p53/PISD signaling contributed to autophagy flux in tumor cells. Besides, by using siRNAs targeting *p53* and *PISD*, we observed that the levels of LC3-II displayed similar tendencies in HCT116 and U-2 OS cells (Figure 4d). In addition, after the p53 level in TFAM knockdown cells was restored by transfection with the pcDNA3.1-p53 construct, both PISD and LC3-II returned to levels similar to those in the control groups (Figure 4e). Since in TFAM knockdown tumor cells, the reduced LC3-II levels were accompanied by decreased expression of p53 and PISD and together with the results that p53/PISD signaling positively contributed to autophagy, we inferred that the attenuated autophagy in TFAM knockdown cells was at least partially attributed to decreased p53 expression and its influence on the transcription of *PISD*.

### 2.5. C-terminal Region Acetylation of p53 Regulates PISD Expression

**Figure 5:**
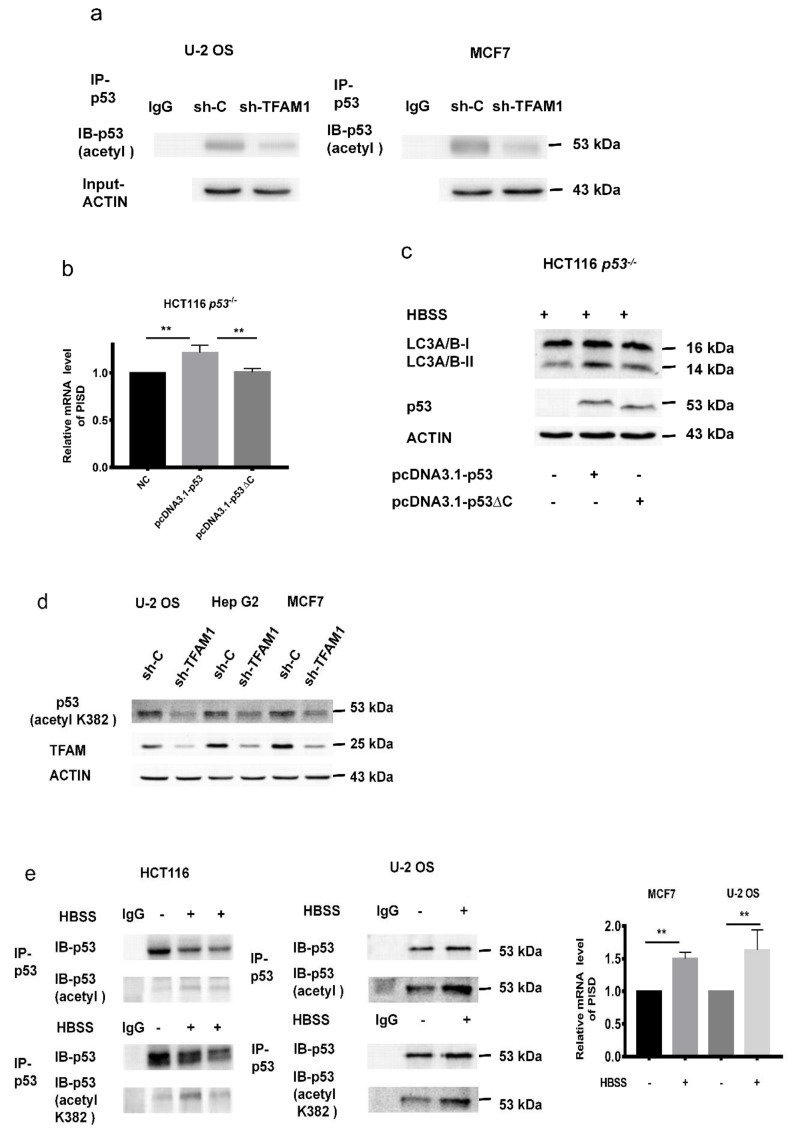
C-terminal region acetylation of p53 regulates PISD expression. (**a**) Analysis of the acetylation of p53 in TFAM knockdown MCF7 and U-2 OS cells after treatment with HBSS for six hours. (**b**) Under HBSS treatment, the mRNA levels of *PISD* in HCT116 *p53^-/-^* cells after being transfected with the pcDNA3.1-p53 or pcDNA3.1-p53∆C construct. (**c**) Under HBSS treatment, the protein level of LC3-II in HCT116 *p53^-/-^* cells after being transfected with the pcDNA3.1-p53 or pcDNA3.1-p53∆C construct. (**d**) The levels of acetylated p53 (K382) in TFAM knockdown cells. (**e**) After treatment with HBSS for six hours, immunoprecipitation and immunoblotting analysis of the acetylation levels of p53 and qRT-PCR analysis of *PISD* mRNA levels in U-2 OS and HCT116 cells. Each independent experiment was repeated three times or more, and data are presented as the mean ± SD. **p < 0.01.

We confirmed that p53 enhanced the transcription of *PISD*, and treatment with pifithrin-α, the inhibitor of p53 transcriptional activity, mitigated the expression of PISD. Since the acetylation of p53 is closely related to the transcription activity of p53, we asked whether the decreased expression of PISD in TFAM knockdown cells was due to attenuated acetylation of p53. Firstly, total acetylation levels of p53 in TFAM knockdown U-2 OS and MCF7 cells were checked by immunoprecipitating total p53 and then probing with acetylated lysine antibody. As shown in Figure 5a, TFAM knockdown resulted in a reduction of acetylated p53. Since the C-terminal region of p53 contains potential acetylation sites, we inserted the p53 coding sequence with 363 to 393 residuals that were deleted into the pcDNA3.1 plasmid (pcDNA3.1-p53∆C). As displayed in Figure 5b, the *PISD* mRNA level in HCT 116 *p53^-/-^* cells transfected with the pcDNA3.1-p53 construct recovered to the level observed in HCT 116 cells after HBSS treatment. However, if the transfected construct was pcDNA3.1-p53∆C, the *PISD* mRNA level did not show an obvious change. Consistently, after HBSS treatment, the LC3-II level in HCT 116 *p53^-/-^* cells transfected with the pcDNA3.1-p53 construct increased, while it remained unchanged if transfected with the pcDNA3.1-p53∆C construct (Figure 5c). We further identified that the acetylation of lysine 382 of p53 (ace-K382) was reduced in TFAM knockdown cells (Figure 5d). Since our results showed that HBSS treatment resulted in increased levels of LC3-II and PISD, we next checked whether HBSS treatment increased the acetylation level of p53. As displayed in Figure 5e, in HBSS treated HCT 116 and U-2 OS cells, the total acetylation level and the ace-K382 level of p53 were notably increased and accompanied by elevated levels of *PISD* mRNA, which were in line with the above findings. Taken together, decreased acetylation at the C-terminal region of p53 in TFAM knockdown cells downregulated PISD and autophagy.

### 2.6. Activation of SIRT1 Inhibits the Acetylation of p53 in TFAM Knockdown Cells

**Figure 6:**
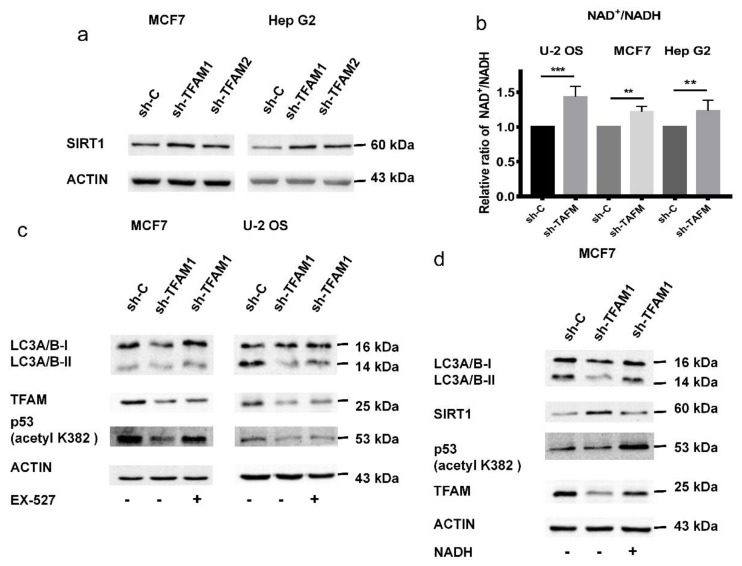
Activation of SIRT1 inhibits the acetylation of p53 in TFAM knockdown cells. (**a**) The protein levels of SIRT1 in MCF7 and Hep G2 cells. (**b**) The ratio between NAD^+^ and NADH in TFAM knockdown cells. (**c**) After being treated by EX-527 (10 µM) for nine hours, the levels of acetylated p53 (K382) and LC3-II were detected in TFAM knockdown cells. (**d**) After being treated by NADH disodium salt (60 µM) for 12 hours, the levels of acetylated p53 (K382) and LC3-II were detected in TFAM knockdown cells. Each independent experiment was repeated three times or more, and data are presented as the mean ± SD. **p < 0.01, ***p < 0.001.

Given that p53 acetylation played an important role in regulating the transcriptional of *PISD* and the levels of C-terminal region acetylation of p53 were decreased in TFAM knockdown cells, we next investigated whether SIRT1, one type of NAD-dependent protein deacetylase involved in regulating the acetylation and stability of p53, participated in the lowered autophagy in TFAM knockdown cells. It was observed that the expression levels of SIRT1 in TFAM knockdown cells were enhanced (Figure 6a). Due to SIRT1 activity being related to the content of NAD^+^ in cells and serving as a sensor of the cytosolic ratio of NAD^+^/NADH, the NAD^+^/NADH ratios in the control and TFAM knockdown cells were measured. As shown in Figure 6b, TFAM knockdown resulted in over a 20% increase in the NAD^+^/NADH ratio, which was consistent with enhanced SIRT1 expression. After being treated by EX-527, a selective inhibitor of SIRT1 activity, TFAM knockdown U-2 OS and MCF7 cells largely had the levels of K382 acetylation of p53 and LC3-II restored (Figure 6c). In addition, after supplementing exogenous NADH disodium salt into the cell culture medium of TFAM knockdown MCF7 to decrease the NAD^+^/NADH ratio, we noted that the expression level of SIRT1 was downregulated, the levels of p53 acetylated at K382 were increased, and autophagy was promoted (Figure 6d).

**Figure 7:**
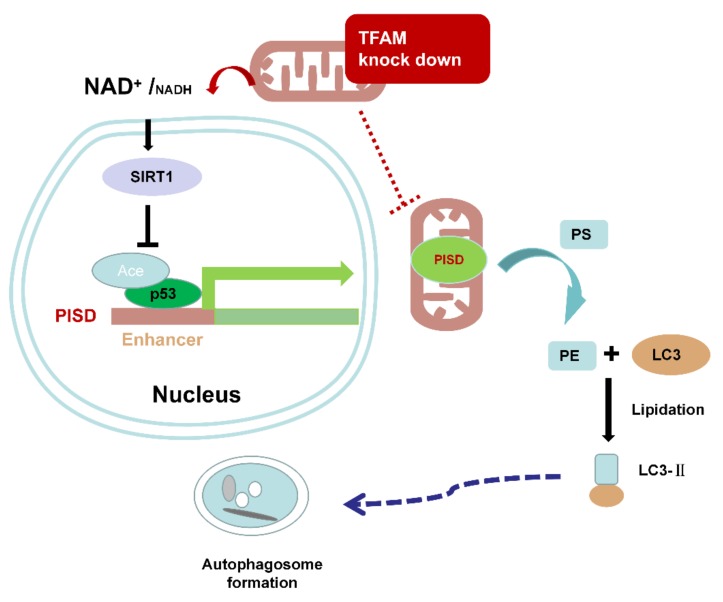
The schematic figure of this study. Downregulation of TFAM increased the NAD^+^/NADH ratio and upregulated SIRT1. SIRT1 deacetylated p53 and resulted in lowered transcription of *PISD*. The attenuated expression of PISD retarded autophagy.

As a summary of this study, downregulation of TFAM increased the cellular NAD^+^/NADH ratio, which led to enhanced expression of SIRT1. SIRT1 stimulated the deacetylation of p53 and resulted in the destabilization and reduced transcriptional activity of p53. This further attenuated the expression of PISD and retarded autophagy (Figure 7).

## 3. Discussion 

TFAM is a regulator of mitochondrial biogenesis and plays an important role in the maintenance of mitochondrial DNA integrity and mitochondrial functions. It has been reported that inhibition of TFAM led to increased sensitivity of cancer cells to cisplatin, doxorubicin, or ionizing irradiation [26,27]. Mitochondrial dysfunctions could result in variations of autophagy. For example, Li et al. reported that the natural contaminant Ochratoxin A damaged the mitochondria of human gastric epithelium cells and triggered autophagic cell death [28]. Yin et al. found that the anti-cancer agent doxorubicin induced cytotoxicity and mitochondrial toxic effects in human adult ventricular cardiomyocyte. At the same time, doxorubicin treatment reduced the expression of mitochondrial proteins and induced mitochondrial autophagy [29]. In the present study, we demonstrated that knockdown of TFAM retarded autophagy flux in cancer cells, which was similar to the finding that in lymphocytes, genetic deletion of TFAM restrained the formation of LC3-II (6). Because of the important role of LC3-II in the elongation and maturation of autophagosome, a change in LC3-II level was an ideal marker to indicate the autophagy influx. Although the loss of LC3 did not prevent the formation of the sealed autophagosome [30], it affected autophagosome size and the efficiency at which they were formed. Besides, lysosomal enzymes selectively degraded the inner autophagosomal membrane, which finally allowed the degradation of the enclosed autophagic content. The degradation rate was suppressed in ATG proteins, which are involved in ATG8 family conjugation deficient cells [31]. Another report showed that in mouse neurons, inhibition of mitochondrial functions, by the deletion of mitochondrial proteins Aif, Opa1, or Pink1, as well as chemical inhibition of the electron transport chain impaired lysosomal activity, which was closely associated with the autophagic clearance of damaged molecules and organelles [32]. Our results clarified that the depletion of TFAM resulted in the downregulation of p53/PISD signaling, which inhibited the formation of LC3-II and retarded autophagy.

PISD is located in the inner membrane of mitochondria and responsible for the production of PE, which is important for mitochondrial lipid metabolism and homeostasis [33,34]. Downregulation of PISD leads to the accumulation of abnormal mitochondria exhibiting disrupted inner membrane integrity and reduced OXPHOS protein content in skeletal muscle [35]. Knockdown or mutation of *Psd* resulted in light-dependent retinal degeneration in the Drosophila visual system [36]. In yeast and mammalian cells, the artificial increase of intracellular PE levels, by provision of its precursor ethanolamine or by overexpression of PE-generating enzyme Psd1, significantly promoted autophagic flux [16]. On the contrary, limiting the activity of PISD and mitochondrial phosphatidylethanolamine impaired autophagy [37]. Our results showed that starvation induced the transcription and expression of PISD coupled with enhanced autophagy. Meanwhile, knockdown of TFAM repressed the expression of PISD and retarded autophagy flux. These confirmed the roles of PISD and TFAM in promoting autophagy flux. 

Our previous study indicated that knockdown of TFAM downregulated the expression of p53. To elucidate how TFAM affected PISD expression, we investigated whether p53 was an upstream regulator of *PISD*. Our results showed that nutlin-3 enhanced, while pifithrin-α inhibited, the transcription of *PISD*. The enhancer reporter assay proved that p53 bound the enhancer region of *PISD* to facilitate its transcription and expression. These data demonstrated that p53 could regulate autophagy through affecting the expression of PISD. Previous studies indicated that p53 can enhance autophagy by directly regulating downstream genes such as *ULK*, *ATG7*, *DRAM*, and *Isg20L1*, which are involved in autophagy [20,38,39]. Besides, several other reports stated that the expression of p53, especially the cytoplasm-located p53 mutants, inhibited autophagy through reducing the phosphorylation of AMPKα, the acetyl-CoA carboxylase α subunit, and TSC2, as well as increasing the phosphorylation of p70S6K [21,40,41]. In general, the subcellular localization of p53 plays a vital role in its regulation of autophagy, and cytoplasmic p53 has a tendency to inhibit autophagy, while nuclear p53 tends to promote autophagy [42]. In this work, we showed that p53 positively stimulated autophagy via enhancing the transcription of *PISD*, in line with the nucleus-localized p53 promoting autophagy. We also found that in p53 null HCT 116 cells, transfection with full length p53 restored autophagy, while transfection with C-terminal deleted p53 could not. The C-terminal region of p53 contains multiple potential acetylation sites [17,43,44]. It was reported that promoting the acetylation of p53 increased its stability and transcriptional activity, which subsequently affected the contribution of p53 to autophagy [39]. Attenuation of p53 acetylation at lys382, which is mediated by CBP/p300, led to the suppression of its target genes [45]. The sequence-specific DNA-binding activity of p53 was dramatically stimulated after the acetylation of its C-terminal domain [46]. The acetylation of the p53 C-terminal domain could modify the duration of the residence of p53 on chromatin [47]. Our results showed that HBSS treatment induced autophagy; meanwhile, enhanced acetylation of p53 and expression of PISD were observed. However, when TFAM expression was knocked down, the acetylation level and stability of p53 decreased, accompanied by reduced PISD expression and retarded autophagy. These indicated that the attenuated acetylation of p53 in TFAM knockdown cells inhibited the autophagy flux.

In this study, we proved that the C-terminal region of p53, especially the acetylation at lysine382 of p53, was important for its regulation of PISD expression and autophagy. Kim et al. showed that lysine382 to arginine mutation of p53 abolished p53-mediated transcription [45]. Ito et al. demonstrated that the acetylation of p53 at lysine382 was commonly induced by multiple p53-activating agents. The maintenance of lysine382 acetylation sustained the stability of p53 [48]. Our results showed that TFAM knockdown attenuated the lysine382 acetylation of p53, associated with decreased stability of p53 and the transcription level of *PISD*. This was in line with the above findings. SIRT1 is a defined p53 deacetylase. It deacetylates lysine 382 of p53 and impairs the capability of p53 to induce transcription-dependent proapoptotic programs and cell senescence [49]. Upregulating SIRT1 expression in cancer cells inactivated p53 and facilitated cells to bypass apoptosis and survive DNA damages [50]. Compound Inauhzin (INZ) can effectively reactivate p53 by inhibiting SIRT1 activity and promote p53-dependent apoptosis in human cancer cells [51]. TFAM was crucial for the transcription of mitochondrial DNA encoded genes. Loss of TFAM impaired mitochondrial oxidative phosphorylation and resulted in excessive accumulation of ROS in cells [52,53]. It was manifested that decreased mitochondrial DNA copy number and increased mitochondrial superoxide level could stimulate SIRT1 activity [54]. We found that TFAM knockdown upregulated the expression of SIRT1. Since SIRT1 is a kind of NAD-dependent protein deacetylase, the NAD^+^/NADH ratios were detected in TFAM knockdown cells. It was observed that the NAD^+^/NADH ratios were enhanced in TFAM knockdown cells and exogenous supplementation of NAD could restore the lysine382 acetylation of p53. These results indicated that metabolic changes associated with TFAM knockdown could affect the expression of SIRT1 and the acetylation modification of p53, leading to retardation of autophagy in tumor cells.

In summary, our present work provided evidence to show that the depletion of TFAM could stimulate the expression of protein deacetylase SIRT1 via enhancing the cellular NAD^+^/NADH ratio. The increased SIRT1 repressed the acetylation of p53, which further resulted in reduced expression of PISD and mitigated autophagy. Our work demonstrated a mechanism for how mitochondrial disfunction regulates cellular autophagy in tumor cells. This might provide additional information on increasing the killing efficiency of tumor cells through regulating mitochondrial functions. 

## 4. Materials and Methods 

### 4.1. Cell Culture 

The human tumor cell lines Hep G2, U-2 OS, and MCF7 were from ATCC (Manassas, VA, USA). HCT 116 and HCT 116 *p53^-/-^* were maintained by our institute. All the cells were cultured in DMEM/F12 supplemented with 10% FBS at 37 °C in a 5% CO_2_ incubator. 

### 4.2. Chemicals and Reagents 

Puromycin, pifithrin-α, and nutlin-3 were obtained from Selleck (Houston, TX, USA). EX-527 and NADH disodium salt were from Solarbio (Beijing, China). Cycloheximide was from Calbiochem (Darmstadt, Germany). The NAD/NADH Quantification Kit was purchased from Beyotime (Shanghai, China). pGL3 Luciferase Reporter Vectors and the Steady-Glo® Luciferase Assay Kit were bought from Promega (Madison, WI, USA). The following primary antibodies were used: TFAM, β-actin, p53, PISD (Santa Cruz, CA, USA), acetylated-lysine, LC3A/B (Cell Signal Technology, Danvers, MA, USA), SIRT1 (Abcam, Cambridge, U.K.). Acetyl-p53 (ZEN BIO, Chengdu, China), HRP-conjugated secondary antibodies were purchased from Jackson ImmunoResearch Laboratories (West Grove, PA, USA). DNA primers were synthesized by General Biosystems (Chuzhou, China). siRNA oligos were synthesized in GenePharma (Shanghai, China) TFAM shRNA was purchased from OriGene (Rockville, MD, USA). 

### 4.3. Transfection of siRNA, shRNA, and pcDNA3.1 Plasmid 

shRNA plasmid targeted human *TFAM* and scrambled shRNA plasmid were transfected into the cells by Roche X-tremeGENE HP DNA Transfection Reagent (Roche Diagnostics GmbH; Mannheim, Germany) according to the manufacturer’s protocol. Medium containing 1 μg/mL puromycin was used to select transfectants. Knockdown of TFAM was confirmed by determining the expression level of TFAM by Western blotting and the mRNA level by quantitative real-time PCR. si-*p53*, si-*PISD*, si-*TFAM*, and pcDNA3.1 plasmid were transfected into cells by Lipofectamine 2000 transfection reagent (Thermo Fishier, Shanghai, China) according to the manufacturer’s protocol. si-RNA sequences are provided in Table S1. 

### 4.4. Western Blotting Analysis 

The cells were washed twice with ice-cold PBS and then lysed with RIPA buffer containing protease inhibitors and protein phosphatase inhibitors. After being incubated on ice for 30 min, the lysate was centrifuged at 12,000 rpm for 10 min at 4 °C. The protein concentration of the supernatant was determined using a BCA kit (Sangon Biotech, Shanghai, China). Protein samples were resolved by 10 or 12% SDS-PAGE and transferred onto a PVDF membrane (Roche Diagnostics GmbH; Mannheim, Germany). The membranes were blocked in TBST (0.1% Tween-20) with 5% non-fat milk at room temperature and hybridized with the appropriate primary antibodies dissolved in TBST containing 5% non-fat milk overnight at 4 °C. After washing three times with TBST, the membrane was hybridized with corresponding HRP-conjugated secondary antibody for 2 h at room temperature and washed another three times with TBST. The membrane was visualized by using the enhanced chemiluminescence substrate (BOSTER Biological Technology, Wuhan, China) in the chemiluminescence image analyzer. Bands’ intensity was analyzed by ImageJ software (NIH, Bethesda, MD, USA).

### 4.5. Immunofluorescence Staining 

To detect the level of autophagy, the cells were washed twice with PBS and fixed with 4% paraformaldehyde at room temperature for 15 min. Then, the cells were permeabilized with 0.5% Triton X-100 at room temperature for 30 minutes and blocked with 1% BSA in PBST (0.1% Triton X-100) at room temperature for 1 h. The cells were then incubated with anti-LC3A/B antibody diluted in PBST containing 1% BSA overnight at 4 °C. After that, the cells were washed three times with PBST for 15 min. Alexa Flour-594 conjugated goat anti-rabbit IgG secondary antibody was used to incubate samples at room temperature for 2 hours. After washing with PBST, cell nuclei were stained with DAPI. Images were captured under an Olympus IX83 fluorescence microscope. Fluorescence intensity was analyzed with ImageJ software (NIH, Bethesda, MD, USA).

### 4.6. Quantitative Real-Time PCR 

Total RNA was extracted using RNAiso (Takara, Shiga, Japan). Real-time quantitative PCR (qRT-PCR) was undertaken using One Step SYBR® PrimeScript^TM^ PLUS RT-PCR Kit (Takara, Shiga, Japan) according to the *ΔΔ^CT^* method. Reverse transcription was carried out at 42 °C for 10 minutes. The primers used for qRT-PCR analysis were: β-actin (forward: CCTGGCACCCAGCACAAT, reverse: GGGCCGGACTCGTCATAC), PISD (forward: ATCACTACCGCAACCTCAGCGA, reverse: TACCTGCTCCACCTCACAGTTC), p53 (forward: CCTCAGCATCTTATCCGAGTGG, reverse: GGATGGTGGTACAGTCAGAGC). The amplification parameters were: 95 °C for 15 seconds, 52 °C for 30 seconds, and 72 °C for 30 seconds. The reaction was performed for 35 cycles. The mRNA level of β-actin was used as the endogenous control. 

### 4.7. The Construction of the Plasmid and the Fluorescence Reporter Assay 

DNA sequences encoding p53 and p53∆C (the carboxyl terminal fragment from residuals 363 to 393 was deleted) were inserted respectively into pcDNA3.1. The obtained constructs were designated as pcDNA3.1-p53 and pcDNA3.1-p53∆C. The predicted enhancer region of *PISD* (NC_000022.11:c31648029-31646950 Homo sapiens chromosome 22, GRCh38.p12) was inserted into the pGL3-promoter vector to construct the pGL3-PISD enhancer reporter plasmid. Then, the reporter plasmid was co-transfected with pcDNA3.1, pcDNA3.1-p53, and pcDNA3.1- p53∆C into HCT116 *p53^-/-^* cells respectively. The luciferase activity was measured using the Steady-Glo® Luciferase Assay Kit (Promega, Madison, WI, USA) according to the manufacturer’s protocol. 

### 4.8. NAD^+^/NADH Assay 

The cells were washed with cold PBS. Then, 2 × 10^5^ cells were harvested by centrifugation at 2000 rpm for 5 minutes and lysed with 400 μL NADH/NAD Extraction Buffer for 20 minutes. After that, the sample was vortexed for 10 seconds and centrifuged at 13,000× g and 4 °C for 10 minutes. The supernatant was collected. Firstly, an appropriate volume of supernatant was incubated at 60 °C for 30 minutes to decompose NAD^+^. Then, the original supernatant (for detection of total NADH and NAD^+^) and the 60 °C treated supernatant were added into a 96 well plate, respectively. After adding the alcohol dehydrogenase working solution, the reaction mixture was incubated at 37 °C for 10 minutes. Then, WST-8 was added into the mixture and incubated 37 °C for another 30 minutes. The absorbance at 450 nm was recorded. The ratio of NAD^+^/NADH in each sample was determined by the corresponding equation.

### 4.9. Immunoprecipitation Assay 

Total cell lysate was prepared using RIPA buffer containing protease inhibitors. After centrifugation, the supernatant was collected. The Protein G agarose bead slurry was added into the supernatant and incubated at 4 °C for 30 min on a rotator. After centrifugation at 1000 rpm for 3 min at 4 °C, the supernatant was transferred to a fresh tube. Primary antibody was added into the supernatant and incubated at 4 °C for 12 hours with gentle agitation. Then, the Protein G agarose bead slurry was added to capture the protein complex. After incubation at 4 °C for 3 hours with gentle agitation, the sample was centrifuged at 1000 rpm for 30 s at 4 °C. The supernatant was discarded, and the pellet was washed with RIPA buffer. Finally, SDS-PAGE loading buffer was used to resuspend the immunoprecipitate for Western blotting analysis. 

### 4.10. Statistical Analysis

Statistical data were expressed as the mean ± standard deviation from at least three independent experiments. Significant differences between two groups were determined by Student’s *t*-test using the GraphPad Prism software (San Diego, CA, USA). For multiple groups comparison, one-way ANOVA analysis by SPSS software (International Business Machines Corporation, Somers, NY, USA) was used. *p*<0.05 represented that the difference was statistically significant.

## 5. Conclusions

Our present work reported that in TFAM knockdown cells, the fluctuation of NAD^+^ and NADH resulted in the activation of SIRT1, which reduced the acetylation of p53. Downregulated C-terminal acetylation of p53 attenuated the expression of PISD, leading to decreased lipidation of LC3 and the retardation of autophagy. Our results provided new data for the understanding of the correlation between mitochondria and autophagy.

## Supplementary Materials

The following are available online at https://www.mdpi.com/2072-6694/12/2/493/s1, Figure S1: The levels of p53 and LC3-II in U-2 OS cells after transfected by siRNA targeted TFAM, Figure S2: The enlarged images of immunofluorescence staining of LC3-II, Table S1: The sequences of siRNA oligos used in this study.
